# Healing of Severe Herpes Zoster Ophthalmicus Within a Few Days: An Autobiographical Case Report

**DOI:** 10.7759/cureus.20303

**Published:** 2021-12-09

**Authors:** Tibor Bakacs

**Affiliations:** 1 Department of Probability, Alfred Renyi Institute of Mathematics; The Eötvös Loránd Research Network, Budapest, HUN

**Keywords:** autobiographical case report, speedy recovery, superinfection therapy, live attenuated vaccine virus, acyclovir therapy, more than three dermatomes, orbital edema, herpes zoster ophthalmicus, shingles, 75 years old patient

## Abstract

Herpes zoster (shingles) is caused by the herpes zoster virus and is characterized by pain and unilateral vesicular rash that typically affects one dermatome. Symptoms tend to resolve over 10-15 days. This case report describes the 75-year-old author’s herpes zoster ophthalmicus (HZO) accompanied by severe orbital edema. The upper eyelid and the proximal nasal area were also affected. The author felt an intermittent throbbing pain in more than three dermatomes including the frontal, orbital, temporal, and occipital/nuchal areas. Since the prodromal and erythematous phase started with atypical signs, conventional acyclovir treatment was administered only 96 hours after the appearance of the first symptoms. Acyclovir treatment was therefore complemented with the experimental viral superinfection therapy (SIT). Superinfection is a host-directed therapy, during which the non-pathogenic avian live-attenuated infectious bursal disease virus (IBDV) vaccine delivers its double-stranded RNA (dsRNA) cargo to host cells and activates their natural antiviral interferon (IFN) gene defense system from within. Most symptoms resolved within five days. Given the author's advanced age of 75 years, such speedy recovery is unlikely to be explained by the belated acyclovir treatment alone.

## Introduction

Herpes zoster (shingles) is a self-limiting condition caused by the reactivation of the varicella-zoster virus (VZV). Shingles are characterized by grouped herpetiform vesicles developing on the erythematous base, pain in the dermatomal area of involvement, which typically affects one dermatome [1]. The risk of VZV increases with advancing age, from 0.86 per 1000 person-years in individuals aged <19 years to 12.78 per 1000 person-years among those aged 80 years and older [2]. In fact, while about one-third of people are affected by shingles during their lifetime, the disease affects one-half of those who live to the age of 80 years. Predisposing factors for VZV reactivation are older age (shingles mostly affect people aged over 60 years), physical trauma (including dental procedures), psychological stress, malignancy, radiation therapy, and immunocompromised states (e.g., transplant recipients, steroid therapy, and HIV infection).

VZV is a neurotropic herpes virus that infects nearly all humans [3]. The primary infection causes chickenpox (varicella). Varicella vesicles can develop on any dermatome. Cell-free VZV has therefore direct access to dorsal root, cranial nerve, and autonomic nervous system ganglia along the entire neuraxis (the axial part of the central nervous system), where VZV becomes latent. Decades after primary infection, as VZV-specific host immunity declines with age, the virus may reactivate spontaneously anywhere on the body. VZV travels from the cell bodies of the neurons to the nerve terminals in the skin characterized by the dermatomal distribution of pain and rash. The course of shingles can be divided into three stages: prodromal stage, infectious rash, and resolution. Early symptoms include pruritus, the involved area being tender to palpation, mild to severe burning sensation, and throbbing or stabbing pain. The prodromal illness lasts one to four days. Then, erythematous macules and papules develop and progress to infectious vesicles that last for 7-10 days. Vesicles initially are clear but eventually become cloudy, ruptured, crusty, and involute. Healing is completed in two to four weeks. While shingles are self-limiting, post-herpetic neuralgia (PHN) is a frequent complication, where pain persists for months or years after the rash has resolved.

When viral reactivation occurs in the cranial nerve (CN) V (trigeminal nerve; Gasserian ganglion), herpes zoster ophthalmicus (HZO) develops [4]. HZO accounts for 10-15% of zoster cases. HZO is characterized by vesicular and erythematous involvement of the CN V1 dermatome, ipsilateral forehead, and upper eyelid. Ipsilateral preauricular (occasionally submaxillary) nodal involvement is a common prodromal event, which together with pain, vesiculation, and erythema is an important symptom in establishing a diagnosis. Development of orbital edema is, however, an ophthalmologic emergency, when patients must be referred immediately for specialized evaluation and treatment. In such cases, iritis, iridocyclitis, glaucoma, and corneal tissue ulcerations may occur.

## Case presentation

**Figure 1:**
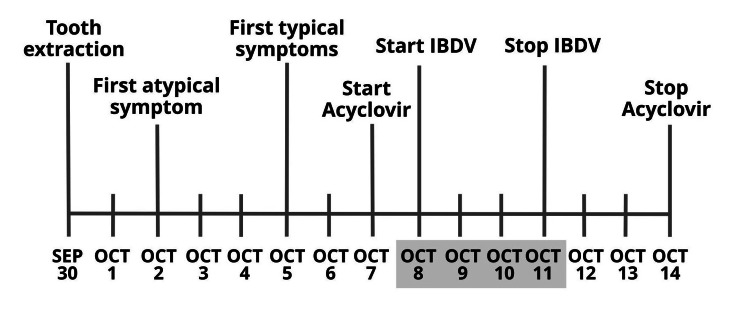
Disease and treatment course Dateline of herpes zoster ophthalmicus and its treatment from September 30 to October 14, 2021. Extended lines represent the start of symptoms and treatment. Days highlighted in gray illustrate treatment with IBDV

I first had varicella at age nine. Now I am 75 years old and this was my first herpes zoster episode. As I have no chronic disease and I do regular physical exercise six days a week, I am in good physical condition. The VZV was probably reactivated by an hour-long tooth extraction that I had (No.8 from the right lower jaw) three days before the first herpes symptoms occurred (September 30, 2021; Figure 1). The prodromal, erythematous, and macular phase was quite atypical at the beginning. I felt only a localized tingling, and itching but not yet a burning sensation at the left side on the top of my head (October 2, 2021). Within a few days, however, the rash became painful and spread to my left face including the periorbital area (October 5, 2021). Of note, the upper eyelid below the palpebral fissure and the proximal nasal area were also involved. By this time, many erythematous papules appeared, which developed into vesicles that began to pustulate and became ulcerated. I felt intermittent throbbing pain on the frontal, periorbital, temporal, and occipital/nuchal areas. The pain was tolerable but it persisted through all the symptomatic stages of my shingles. I also had systemic symptoms, including malaise, fever, and headache. An ipsilateral preauricular lymph node became palpable as well.

Due to the atypical mild syndromes during the first few days, I did not recognize that I had shingles and therefore started conventional standard acyclovir treatment belatedly, 96 hours after the onset of first symptoms (October 7, 2021) (Egis Pharmaceuticals PLC, Budapest, Hungary; 5x800 mg daily, for seven days). However, when the rash and vesicles expanded to the periorbital region including my upper palpebra with fast-increasing edema around my eye accompanied by strong yellow discharge, I realized that I had HZO. Importantly, the neuralgia affected the ophthalmic (V1), maxillary (V2), lesser occipital (ventral ramus C2), and greater occipital (dorsal rami C2 and C3) dermatomes, respectively. As the posterior scalp (nuchal area) was involved in the neuralgia, herpes occipiticollaris was also suspected. Herpes involving all three branches of the trigeminal nerve is known as disseminated zoster and seen in immunocompromised patients but is a rarity in immunocompetent individuals [5].

**Figure 2:**
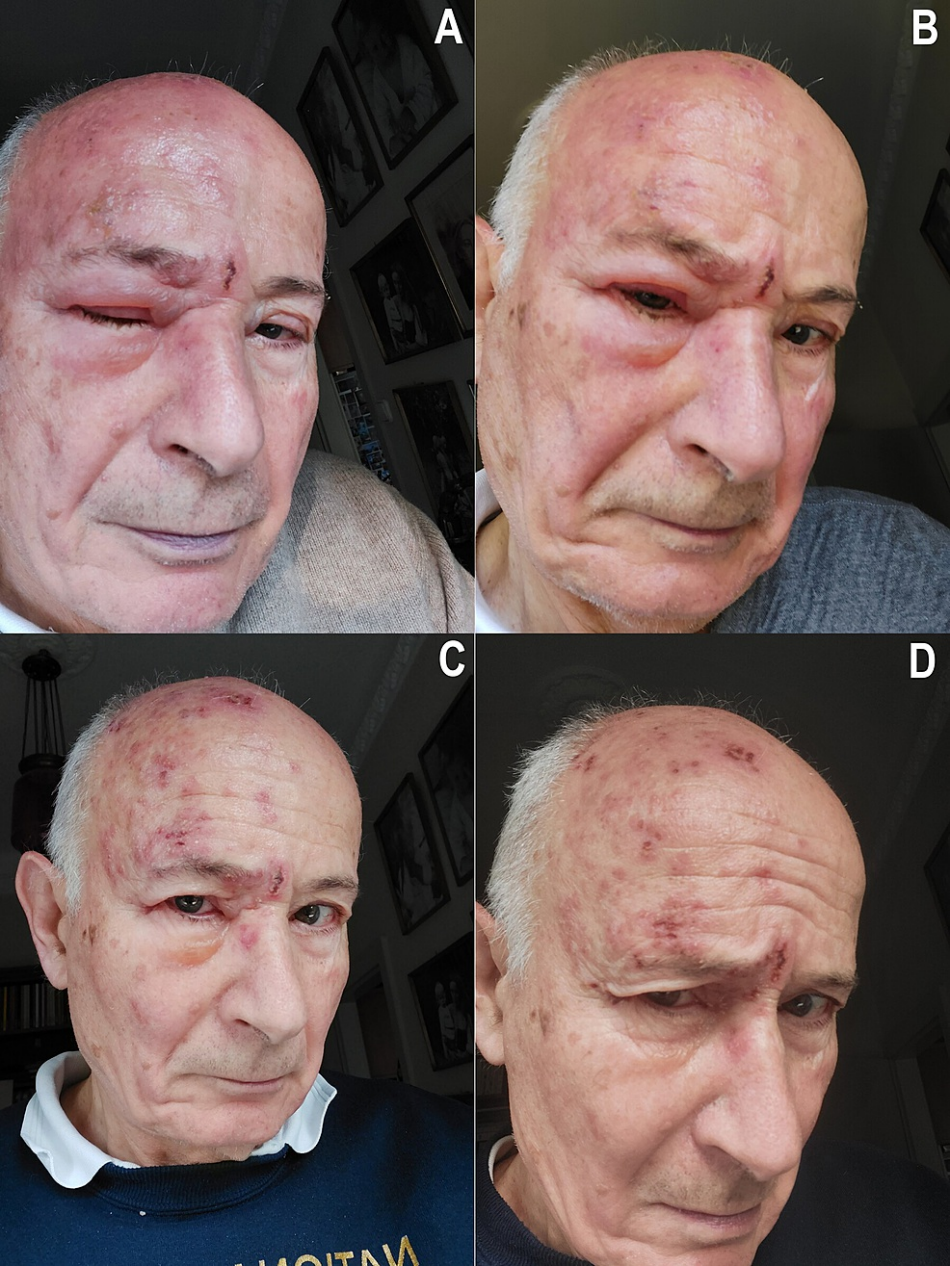
The author's HZO with orbital edema at the peak of disease and in recovery (A-D) The selfie pictures were taken between October 9, 2021, and October 12, 2021. Consent to the publication of patient information was granted by Tibor Bakacs, M.D., Ph.D., D.Sc., as he was the patient *and* the treating physician in this autobiography.

I am the Chief Scientific Officer (CSO) of a company that develops broad-spectrum viral superinfection therapy (SIT) for the post-infection treatment of viral diseases. SIT is a host-directed therapy during which the non-pathogenic avian live-attenuated infectious bursal disease virus (IBDV) vaccine delivers its double-stranded RNA (dsRNA) cargo to host cells and activates their natural antiviral interferon (IFN) gene defense system from within [6,7]. Since I started acyclovir treatment too late, I complemented it with SIT (October 8, 2021). Although our company has no experience with herpes zoster yet, based on our prior investigations demonstrating that oral treatment with the IBDV is safe and potentially effective for the treatment of other viral infections, I have orally taken avian live-attenuated IBDV vaccine, 2x10^6^ IU/day for three days [10^6^ IU = 10^6^ TCID_50_ (TCID = tissue culture infective dose)], and then 10^6^ IU/day for one more day (7x10^6^ IU in total). The orbital edema rapidly decreased by IBDV treatment and crusting of the ulcerated vesicle started. Eventually, the rash almost completely subsided by October 12, 2021 (Figures 2A-2D show my HZO from its peak to the recovery from the disease.).

A slit-lamp examination by an ophthalmologist excluded corneal tissue ulcerations. On October 18, 2021, I restarted IBDV treatment (10^6^ IU/day) for seven days (7x10^6^ IU in total) in order to mitigate PHN, which is observed in well over 50% of patients with HZO and can be severe and long-lasting [5]. Three weeks after the last IBDV dose, my serum contained anti-IBDV neutralizing antibodies, confirming the results obtained in animal models that following oral administration of IBDV, neutralizing antibodies were induced. Humans are not a natural host of IBDV. Therefore, the appearance of neutralizing anti-IBDV antibodies demonstrates the interaction of IBDV with the immune system. At the time of writing (as of November 20, 2021), I still have mild PHN in one dermatome. It is tempting to speculate that IBDV treatment also shortened the duration of PHN. The only adverse event of SIT was a slight rise in temperature (up to 37.5 °C) following the oral administration of the IBDV, which has been reported in other patients as well.

## Discussion

Early diagnosis and prompt treatment of shingles in the prodromal phase by anti-viral agents are associated with high efficacy and favorable outcomes [8]. In most individual studies of herpes zoster, the time required for the vesicles, ulcers, and crusts to resolve was shorter when antiviral medication was used compared to placebo, but only by one or two days [9]. Quicker healing of skin lesions was not observed. The efficacy of acyclovir treatment (800 mg, five times daily, for seven days) initiated more than 72 hours after the onset of skin rash has, however, never been confirmed. Furthermore, healing may take longer in older patients. Antiviral medicines may still be considered up to seven days after the onset of symptoms if the patient has an increased risk of severe disease due to his/her older age or life-threatening complications, such as ophthalmic involvement or PHN. I, therefore, started acyclovir treatment despite the fact that the first symptoms had occurred 96 hours earlier. For the very same reasons, I complemented the conventional treatment with our experimental viral (IBDV) SIT even though we have never tested IBDV for the treatment of herpes zoster.

Most viruses produce dsRNA at some point during their replication cycle; dsRNA is a molecular pattern that is associated with viral infection [10]. Attenuated IBDV vaccine strains genomes are composed of dsRNA. Activation of the interferon gene defense system is induced via Toll-like receptors (TLRs), which are a family of innate immune-recognition receptors that recognize molecular patterns associated with microbial pathogens. IBDV has been previously used to successfully treat hepatitis A virus (HAV) infection in marmoset monkeys and in patients with acute and chronic (decompensated) hepatitis B virus (HBV) and hepatitis C virus (HCV) infection [11-13]. Importantly, the patients with decompensated liver disease had high-level viremia, which is one of the key drivers of a cytokine storm. IBDV treatment, however, did not induce the excessive release of pro-inflammatory cytokines.

**Figure 3:**
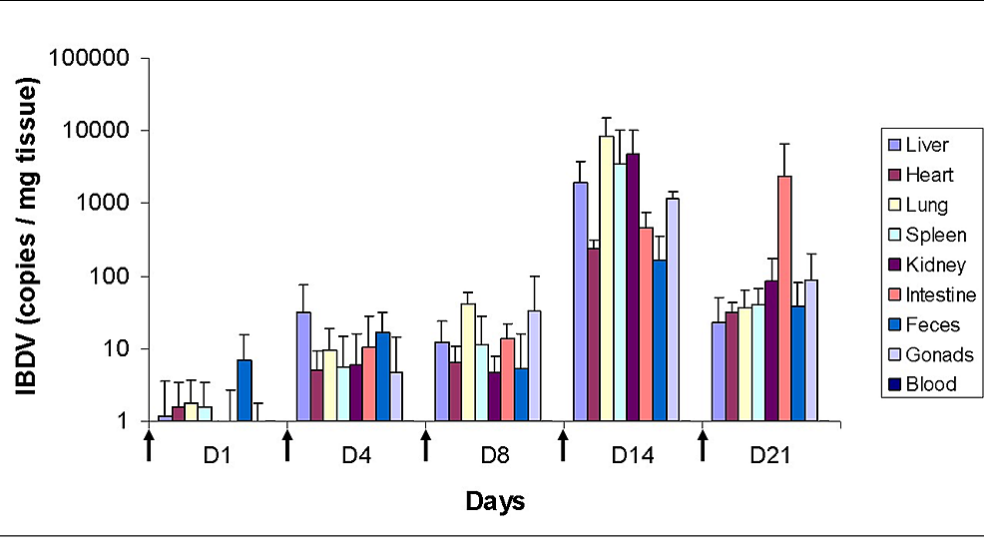
Tissue distribution of R903/78 virus following multiple oral administrations in mice Balb/C mice were dosed with multiple oral deliveries of 1.7x10^6^ IU of IBDV on days 0, 3, 7, 13, and 20 (indicated with arrows). Necropsy was performed on days 1, 4, 8, 14, and 21. Tissue RNA was quantified by quantitative real-time RT-PCR. The transduction efficiency is expressed as IBDV copy numbers per mg of tissue. Each bar represents the arithmetic mean of four independent experiments, except D21 where 1 mouse died. Error bars indicate the standard error deviation (±SD; n = 4; n = 3 for D21) Reproduced with slight modification from Hornyak et al., J Gene Med 2015, 17, 116 [15] with permission from John Wiley & Sons, Ltd. (License Number: 5176880126861)

The safety of SIT is in sharp contrast with the toxicity of systemic IFN-based therapy that causes significant morbidity requiring dose reduction or even discontinuation of the treatment [14]. A possible explanation for this difference could be the contrasting target ranges of the two therapies. Systemic IFN therapy has the almost ubiquitous characteristic of broad immune signaling because receptors for type I and type II IFNs are found on the surface of most human cells. In contrast, following host-cell exposure to IBDV, its dsRNA is recognized by specific receptors (e.g., TLR3), which activate several gene families from within [7]. In this context, it is important to recall that we investigated the biodistribution of the IBDV following multiple oral administrations in mice, which was assessed in multiple organs using real-time quantitative polymerase chain reaction (qRT-PCR) [15]. Despite the presence of high levels of neutralizing antibodies, there was a substantial increase in virus copy numbers in most organs. The highest amounts of virus RNA were detected in the liver, spleen, lung, and kidney (Figure 3).

These data indicated viral stability and genome accumulation under the multiple dosing scenario rather than viral replication in mouse tissues. This is consistent with clinical observations in decompensated hepatitis patients [14] when large doses of the viral preparation were administered continuously over a long period to ensure maintenance of "artificial viremia" by IBDV, which is not known to infect human beings naturally. We have not investigated the presence of the virus in the nervous system yet. The current clinical observation would however encourage the extension of the biodistribution study to the cell bodies of neurons and the skin.

The proof-of-principle of SIT was recently demonstrated in one patient with early coronavirus 2019 (COVID-19) disease [16]. Two additional COVID-19 patients have responded similarly to oral treatment with IBDV (unpublished results).

IBDV is simple to manufacture and can be affordable even in resource-limited countries. Acid-resistant IBDV can be orally administered in an outpatient setting, providing the greatest ease of dosing and the highest chance of patient compliance. The Paul Ehrlich Institute in Germany supports a phase I safety study for persons acutely infected with severe acute respiratory syndrome coronavirus 2 (SARS‑CoV‑2). An expert team of the US National Institutes of Health-sponsored ACTIV public-private partnership came to the conclusion that the IBDV drug candidate shows merit as a potential treatment for COVID-19 (personal communication from Joseph P. Menetski, Ph.D. Associate Vice President, Research Partnerships Foundation for the National Institutes of Health, Inc.). An FDA-approved clinical COVID-19 trial is in its preparatory stage in Los Angeles.

As I am 75 years old and started acyclovir treatment 96 hours after the onset of the first symptoms, I consider my speedy recovery most unanticipated. To this end, I have requested a second opinion from two colleagues who have several decades of experience with herpes zoster.

Steve Bukosza, MD, wrote that during his close to 50-year internal medicine practice, he treated hundreds of patients with malignant and non-malignant diseases suffering from symptomatic herpes zoster. In his experience, the entire disease course usually spanned five to six weeks, in which the ulcerous phase was the longest (more than two weeks). Acyclovir administered even at the onset of the earliest symptoms could only modify the symptoms but was unable to change the duration of the disease course. In this context, the duration of the author’s case seems to be unusually short. The onset of vesicles was typical but the severe orbital edema was an early sign of the severity of the HZO. In contrast, the fast resolution of the orbital edema and the very quick crusting of vesicles cannot be explained by the acyclovir treatment alone. Dr. Bukosza has never seen such a speedy recovery from facial herpes zoster before. He has attributed the efficacy of the treatment and speedy recovery to the experimental SIT.

Professor Shimon Slavin, who was the Director of the Department of Stem Cell Transplantation & Cancer Immunotherapy of the Hadassah Medical Center in Jerusalem for 30 years, wrote that the speedy recovery of the author from HZO was indeed remarkable because specific treatment was started late. Since herpes zoster tends to be more severe in immunocompromised patients, including cancer patients treated with chemotherapy and even more so in patients following allogeneic stem cell transplantation, trying the IBDV treatment for herpes zoster infection in patients at risk seems to be highly desirable. Notwithstanding, only a prospective randomized study will confirm whether the combined IBDV and conventional anti-herpes zoster medications will facilitate recovery compared to conventional monotherapy. In fact, in two cases involving elderly patients with facial herpes zoster (without HZO) who were treated with valacyclovir and acyclovir, respectively, complete regression was observed after two weeks [5].

While this case report lacks statistical comparison, it can provide individual clinical insights that are missed in clinical trials [17]. Breakthrough cases have paved the way for revolutionary medical advances, such as, for example, the first cord blood (CB) transplant, which was performed in 1988 in a patient with Fanconi anemia [18]. Since then, CB transplantation has become standard therapy. Similarly, ever since the experimental chimeric antigen receptor (CAR) T cell therapy resulted in curing an advanced leukemia patient, it has become a promising treatment modality [19].

## Conclusions

The 75-year-old author’s severe HZO with orbital edema completely subsided within a few days by acyclovir treatment in combination with the experimental IBDV SIT. As the acid-resistant IBDV can be orally administered in an outpatient setting, it provides the greatest ease of dosing and the highest chance of patient compliance. IBDV is simple to manufacture and can be affordable even in resource-limited countries. The author’s unexpectedly speedy recovery from HZO infection will hopefully generate a debate about the potential application of IBDV for the adjuvant treatment of severe herpes zoster, including HZO.
